# Towards a More Evidence-Based Risk Assessment for People in the Criminal Justice System: the Case of OxRec in the Netherlands

**DOI:** 10.1007/s10610-022-09520-y

**Published:** 2022-07-01

**Authors:** Seena Fazel, Amir Sariaslan, Thomas Fanshawe

**Affiliations:** 1 Department of Psychiatry, University of Oxford, Warneford Hospital, Oxford, UK; 2 Nuffield Department of Primary Care Health Sciences, University of Oxford, Oxford, UK

**Keywords:** Risk assessment, Violence, Recidivism, AUC, Calibration, Prediction

## Abstract

Risk assessment tools are widely used throughout the criminal justice system to assist in making decisions about sentencing, supervision, and treatment. In this article, we discuss several methodological and practical limitations associated with risk assessment tools currently in use. These include variable predictive performance due to the exclusion of important background predictors; high costs, including the need for regular staff training, in order to use many tools; development of tools using suboptimal methods and poor transparency in how they create risk scores; included risk factors being based on dated evidence; and ethical concerns highlighted by legal scholars and criminologists, such as embedding systemic biases and uncertainty about how these tools influence judicial decisions. We discuss the potential that specific predictors, such as living in a deprived neighbourhood, may indirectly select for individuals in racial or ethnic minority groups. To demonstrate how these limitations and ethical concerns can be addressed, we present the example of OxRec, a risk assessment tool used to predict recidivism for individuals in the criminal justice system. OxRec was developed in Sweden and has been externally validated in Sweden and the Netherlands. The advantages of OxRec include its predictive accuracy based on rigorous multivariable testing of predictors, transparent reporting of results and the final model (including how the probability score is derived), scoring simplicity (i.e. without the need for additional interview), and the reporting of a wide range of performance measures, including those of discrimination and calibration, the latter of which is rarely reported but a key metric. OxRec is intended to be used alongside professional judgement, as a support for decision-making, and its performance measures need to be interpreted in this light. The reported calibration of the tool in external samples clearly suggests no systematic overestimation of risk, including in large subgroups.

Risk assessment tools are widely used at different stages at the criminal justice system, as discussed in this special issue on their use in sentencing, and can assist with decisions about treatment allocation, the level of supervision, and need for additional risk management strategies (Monahan & Skeem, 2016). These tools can vary from simple unweighted scales that rely only on criminal history factors, to more complex statistical algorithms that draw on socio-demographic, psychological, and contextual factors. Generally, these tools are not intended to determine decisions by criminal justice professionals. Rather, they are intended to assist and inform such professional judgements, which are necessarily guided by additional individual factors that risk assessment tools will not be able to capture. Such professionals typically undergo relevant training, and their experience and supervisory framework will also inform their decisions. In addition, all such decisions will have to be made within any regional and national legal structures that may apply, such as sentencing guidelines or what type of supervision is available.

The usefulness and limitations of risk assessment tools in criminal justice has been widely discussed, including their potential risks of exacerbating racial or ethnic biases (Dressel & Farid, 2021). However, important considerations that argue in favour of their use include improving consistency and transparency in risk decisions and risk communication, and potentially anchoring risk assessment in the highest quality empirical evidence. This may mitigate against biases in human decision-making, which may include racial, age, social class, mental health, or gender-based ones. At the same time, risk assessment can be associated with other problems, and this commentary will discuss these and how OxRec (Fazel et al., 2016), a new risk assessment tool that has been implemented by probation services in the Netherlands (Fazel et al., 2019), has addressed them. As part of this, we will address whether the use of data on neighbourhood deprivation can potentially improve risk predictions without contributing to increased racial or ethnic discrimination.

## Problems with Existing Risk Assessment Tools

Much of the legal and sociological commentary on these tools has focused on their potential biases. However, there are some other methodological and practical problems with most existing risk assessment tools, which may lead to no improvement in crime outcomes and can also contribute to producing biassed outputs. First, they can be resource intensive and divert the attention of criminal justice and healthcare staff from the more important risk management activities. For example, a widely used tool in forensic mental health is the HCR-20. Its first use was estimated to take 16 person-hours to complete (Viljoen et al., 2010), partly as it requires a multidisciplinary approach and also additional interviewing for the psychological part of the assessment. The OASys tool, which is an actuarial tool used in England and Wales, consists of more than 100 items (Howard & Dixon, 2012). Part of the justification for these long and complex tools is that they identify needs, which can form the basis of risk management. The problem with this is that predictors and needs are not interchangeable, and a tool that includes many needs will not necessarily result in an improvement in its predictive performance, and doing so may detract from its predictive accuracy. For example, it has been consistently found that the best predictors of crime and repeat offending are calendar age and sex (Chang et al., 2015; Sariaslan et al., 2020), which are not needs. Tools such as the HCR-20 do not include either of these, and the STATIC-99, which is commonly used to assess sexual offending risk, does not include sex and uses four broad age bands (rather than calendar age, which would allow for considerably more precision). The resource intensiveness of risk assessment tools mostly applies to structured clinical judgement (SPJ) tools, while, in contrast, some actuarial tools are quick to use.

Second, some tools insist on a system of paid-for regular training, which can be run by companies that are owned by the tool developers. Training may in fact be necessary to use such tools optimally, but it adds to the economic costs of their use. With the costs already incurred in the time taken to complete the more complex commercially available tools, these further costs of training are an important consideration for criminal justice agencies. Choices about which tool to use will require these expenditures to be balanced against evidence of their predictive performance and ultimately reduction in criminal behaviour (Senior et al., 2020).

Third, many of these older tools are based on dated and suboptimal methods, which are known to overestimate performance and not validate well in new populations. These methods have been improved in other branches of science, particularly in cardiovascular and cancer medicine, where prediction models are widely used and linked to evidence-based treatment allocation (Andersson et al., 2019; Arnett et al., 2019). Key elements include prespecifying predictors in the development of a tool, the outcomes, the statistical methods, and performance measures (Collins et al., 2015). In addition, any tool should be externally validated in a new sample, and where that is not possible, some evidence of internal validation should be presented using methods such as cross-validation or bootstrapping. The latter is an internal re-sampling technique where the original sample is used to create several test samples by randomly selecting the participants with replacement (e.g. the first participant not being selected while the second being selected twice for a given test sample) (Efron & Tibshirani, 1994). By testing the model performance of the tool across the generated samples and pooling the results, the bootstrapping approach allows for an estimation of performance statistics for the tool. The number of outcome events relative to the number of predictors used should be large enough to prevent excessive overfitting (a term used in statistics to refer to the model being too closely aligned with the underlying data, thus not being able to accurately predict the outcome of interest in new samples and populations). A simple rule of thumb is that there should be at least 10 outcome events (i.e. instances of crime), and preferably more, for each predictor tested in any development sample, and at least 100 outcome events in an external validation sample (Collins et al., 2016), which can be more when applying certain machine learning methods. The different risk factors that are used in any tool should be tested in multivariable models (so that the risk factors are mutually adjusted for), and the form of the model, such as a prediction equation, should be made available so that the effect sizes of specific risk factors on criminal outcomes can be seen. Such basic requirements are rarely done in older tools. For some, it is not clear how the original set of risk factors were chosen. For others, the predictors are based on old systematic reviews, such as the 1998 one by Bonta, Law, and Hanson (Bonta et al., 1998), which may have informed the choice of predictors and their weighting of some previous tools (such as the Violent Risk Appraisal Guide [VRAG], where a diagnosis of schizophrenia is negatively scored). It has been shown that the 1998 review combined samples inappropriately for the risk factor of psychosis (or severe mental illness) (Fazel & Yu, 2011), which the original review found to be protective for repeat offending, whereas subsequent and replicated work has found that psychotic disorders are associated with increased risk (Whiting et al., 2021). These methodological shortcomings apply equally to actuarial and SPJ tools (Fazel et al., 2022).

The fourth problem with many tools is that their included risk factors and risk markers are not consistent with the latest empirical evidence. Psychosis, which is a protective factor in the VRAG, is one example cited above. Another is ‘young age of first violent incident’, which is included among the 10 static factors in the HCR-20-v2. A study looking at more than 30 background criminal history factors found that the age of first offence was the least correlated with future crime in a sample of 13,806 people with schizophrenia—the strongest being previous violent conviction (Witt et al., 2015). HCR-20-v3 has changed this to under 18, but this variable also has effect sizes smaller than many other criminal history variables (Witt et al, 2015). Although using existing published research as a guide to deciding which risk factors to include in prediction models is regarded as good practice, this is only beneficial if that research is up-to-date and of high quality and reviewed in a systematic, comprehensive, and transparent way.

Fifth, there are a group of tools used in criminal justice that allow for professionals completing them to decide whether someone being assessed is high, medium, or low risk, without any scoring of background risk markers. These are called structured clinical judgement (SPJ) tools, and they have been popular over the last decade. In other words, a series of questions about risk factors is completed, and on the basis of this, a decision is made on the risk category without a clear sense of how each risk category relates to a likelihood or range of likelihoods of future risk. For example, if two individuals have identical risk factors, one might end up being classified as medium risk and the other as low risk, largely on the basis of how the assessor interprets the relative importance of these risk factors. A related problem is that the risk factors in these SPJ tools typically allow for each risk factor to be scored on a 2-point scale on the basis of how serious or relevant it is, which can lead to a lack of reliability between rates (i.e. inter-rater reliability). Importantly, these risk categories (such as low/medium/high) are not correlated with actual risk as defined by the percentage of criminal or violent outcome events that subsequently occur. Consistent with this, a systematic review found that a ‘high risk’ classification for the HCR-20 was associated with actual annualised violence rates ranging from 5 to 100%. Even for those tools with defined categories, these were poorly calibrated, so that scores of 14 and more for the VRAG corresponded to a range of 7 to 75% violence in the year after assessment. This suggests that the risk categories in older risk assessment tools have little, if any, practical utility for prediction.

A sixth important concern, on which legal scholars have commented widely, is that there is a lack of transparency in how these tools actually come up with their scoring systems, including the threshold for different cut-offs (e.g. ‘low’, ‘medium’, and ‘high’ risk). In many cases, this is because of commercial interests and the tool developers not wanting to share their algorithms. The lack of transparency cannot solely be attributed to commercial interests as public institutions commonly implement these tools without making sufficient demands for transparency, which is important for validation purposes (Carlson, 2017; Feeley, 2002). This may further lead to embedding various biases, including racial or ethnic ones (see below), lack of public trust in their use, and difficulties for criminal justice professionals in understanding how individual risk factors contribute to the risk scores that are ultimately calculated. It may also mean that independent researchers cannot examine these tools, which is necessary to establish their external validation.

A different set of criticisms have been offered by other legal scholars, who have pointed out that algorithms can act to simply provide a veneer of legitimacy. These scholars highlight the lack of their transparency in their use (Lynch, 2019), including poor implementation practices (Stevenson, 2018). In other words, what is not clear in a judicial process is the weight such tools are afforded in decision-making and the impact this has on individuals in the criminal justice system (Hannah-Moffat, 2013). Wider criticisms about how they distort justice by exerting iatrogenic effects on those being assessed (Harcourt, 2008) and undermine fairness (Starr, 2014) are important to consider and should be subjected to rigorous empirical tests. In mental health settings, specific violence outcomes have been examined using trial methodology (Abderhalden et al., 2008; Troquete et al., 2013). In prison and probation settings, the trial approach to empirically testing potential benefits and harms is much less prevalent and needs to be prioritised in future research.

## Racial or Ethnic Profiling

The American debate has been dominated by the possible racial or ethnic profiling associated with some risk assessment tools (Rudin et al., 2020). It has been argued that, even if race or ethnicity is not explicitly considered during risk assessment, some factors in these tools are so correlated with race or ethnicity that they act as proxies. This is made all the more difficult if such tools do not publish how each risk factor is weighted towards the overall risk score. However, although there is some evidence in support, it is not clear. First, what is being compared? If the alternative is to remove any tools, this may lead to human decision-making that is less accurate, more biassed, and even less transparent (Desmarais, 2020). If human-decision-making is biassed and inconsistent, which has been clearly replicated in different settings by behavioural scientists and also in medicine (Saposnik et al., 2016), then an approach that improves the transparency of the assessment process, even if it cannot eliminate all limitations of the existing process, is surely preferable. Second, it needs to be empirically shown what factors might act as proxies. Research has shown, for example, that using historical arrests is problematic but this is not the case when using more severe criminal history variables, such as violent crime convictions (Skeem & Lowenkamp, 2016). Third, the debate needs to move beyond ‘the shadow of COMPAS’ (Desmarais, 2020)—COMPAS is a commercially available algorithm to assess recidivism risk, and only one of the many hundreds of risk assessment tools, used solely in some part of the USA, and its particular problems are not shared with most risk assessment tools. COMPAS lacks transparency, but criticisms of its performance, such as the study by Dressel and Farid (2018), are also problematic because of the way they measured the outcome (yes/no) which is not how such tools are used. Most actuarial tools provide a probability score rather than a simplistic dichotomous classification (Holsinger et al., 2018). Third, there is the wider question about the choice of what variables to include and the implications of excluding variables for the accuracy of a risk tool. Balancing the ethical issues needs to be openly discussed by researchers. In addition to the possibility that these tools may embed structural and systemic biases, ethical questions include the degree of transparency in how the different factors collected by these tools generate predictions and how the outcomes of these tools are actually used in practice, their effects on decision-making, and the consequent effects on the legitimacy of procedural justice and open justice (McKay, 2020). An important consideration is that new tools should be developed on racially or ethnically diverse samples in order to be relevant to the real world, unlike some risk assessment tools that have been developed on white-only samples (Hannah-Moffat & Struthers Montford, 2019).

## Neighbourhood Deprivation Indicators

It has been argued that residence in a deprived neighbourhood can act as a proxy for race or ethnicity. Stating this as a possibility is of course different from having evidence in support. Three considerations are important in this regard. First, what constituents make up a variable of ‘neighbourhood’? Second, what is its contribution to the overall risk score? Third, is there any evidence that a tool which uses neighbourhood-level measures systematically leads to higher overall risk scores for some subgroups?

The OxRec tool was developed using an eight-component neighbourhood deprivation score (Sariaslan et al., 2013), which was converted into deciles for the purposes of the tool. The increase in risk per decile of neighbourhood deprivation score was very small: a hazard ratio of only 1.03. By comparison, the hazard ratios for male sex and drug use disorder were 2 and 1.5, respectively. Adjusting for immigrant status, which might be considered another possible proxy for race or ethnicity, albeit again an imperfect one, did not change the effect size of the neighbourhood in the Swedish development, and this adjusted hazard ratio (i.e. independent of immigrant status) was used. These considerations, when taken together, substantially reduce concerns that neighbourhood may be acting as a simple proxy for race or ethnicity. At the same time, the immigrant variable in the Dutch version of the OxRec was not used. Third, calibration plots were reported where estimated scores compared to predicted scores can be seen in the large Dutch validation samples of released prisoners and people on probation (Fazel et al., 2019). There is no evidence in either sample that there is any systematic overestimation of risk—if anything, there is an underestimation of risk at the higher scores. To be certain if there is any underestimation or overestimation of subgroups by race or ethnicity, calibration could be tested in this way. Nevertheless, as racial or ethnic minority groups make up a large subgroup of people in the sample, then any systematic miscalibration could be observable in the calibration plots, which it is not. Fourth, OxRec was developed in Sweden, and the extent of degree of systemic racism in their legal system is an empirical question that has not been thoroughly addressed (Bäckman et al., 2021). The only register-based study published to date had weak controls for confounding factors and did not find a consistent pattern of discrimination based on immigrant background in the Swedish legal system (Kardell, 2006). Finally, there is the wider issue of including static variables, which some consider lead to ethical problems as they are either not possible or very difficult to modify. It has been discussed elsewhere that these could be included if they add to predictive accuracy (Douglas et al., 2017) but as long as the evidence is good in support of their incremental value. Recent contributions to this debate are notable (Ryberg, 2020) and the importance of weighing up consequentialist calculations with ethical ones. In relation to neighbourhood, however, it is not necessarily the case that it is a static variable—including it in risk tools can act to underscore its importance from public policy and health perspectives and lead to community-based initiatives to reduce neighbourhood deprivation.

## OxRec in the Netherlands

OxRec addresses many of the main limitations of previous risk assessment tools. It was developed a large and non-selected population of released prisoners and then validated externally in a separate population in Sweden and also in the Netherlands. Its development and validation used high-quality methods that meet current recommended methodological and reporting standards (Collins et al., 2015). These include prespecifying the predictors and outcomes, testing predictors in multivariable models, and reporting a full range of performance measures, including measures of discrimination (how well the tool separates individuals who offend from those that do not) and calibration (how close the expected probability scores are to the actual criminal rates). In addition, OxRec is based on predictors that are easy and simple to score and do not require additional interviewing. OxRec typically takes 10–15 min to complete and does not require face to face training—a short video provides some explanation of how it works. Moreover, it was provided free to the Dutch probation service as the developers are university academics working in public universities who were funded by a charity (Wellcome Trust, 2021). The study protocol, all the coefficients and the formula for the final risk score are published (Fazel et al., 2016). It is, to our knowledge, the most transparently reported risk assessment tool to date.

Importantly the OxRec studies tested and reported calibration, which is missed in previous risk assessment studies (Fazel et al., 2022; Van Calster et al., 2019). OxRec outputs, which include 1- and 2-year probabilities of violent and any reoffending, are more informative than using categories such as low, medium, and high, even if those categories have cut-offs assigned to them. For example, it is possible for a tool to discriminate well on whether someone is high risk or low risk using a particular cut-off, while the numerical value (i.e. probability of repeat offending) of the risk prediction is systematically off target. Numerous factors could potentially cause poor calibration in an external validation sample, including differences in participant characteristics and outcome prevalence rates compared with the tool’s development sample. This is important to test and report in risk assessment research because a poorly calibrated tool can overestimate the score of those at higher risk, for example, scoring them all over 60% when in reality their actual offending rates are in the range 30–50%.

In terms of model discrimination, the first external validation of the OxRec, which was conducted in Sweden, reported an overall area under the curve (AUC), or c-statistic, of 0.76. The AUC is an overall measure of discrimination, but it does not capture the balance between false positives and false negatives (Mallett et al., 2012), which are important in criminal justice (where false negatives have been argued to be more problematic than false positives from a criminal justice perspective; false positives can be tolerated if associated with non-harmful outcomes such as more supervision and psychosocial interventions) (Pickard & Fazel, 2013). In the Dutch validation, the AUC was reported to be 0.68–0.69, depending on the population and follow-up period (Fazel et al., 2019). It should be noted that this represents a probable floor on the performance as, in this retrospective validation, some proxy variable had to be used if data on predictors were unavailable, whereas in practice, more accurate risk factors will be collected. Furthermore, the question of whether this AUC is sufficiently high for use in the criminal justice system needs to consider calibration (which is good) and also the fact that OxRec is used as a decision-making support. The empirical question is whether the predictive performance of probation officers in the Netherlands using OxRec with the other tools that are also part of the assessment process, and in the context of their own training and experience, is good enough and how it can be improved.

The process for implementing a tool would ideally include external validation, which should aim to be adequately powered (i.e. have more than 100 violent reconvictions), use predictors from the tool rather than proxy markers, and be conducted across different types of criminal justice settings (depending on the intended use of the tool). This should then be followed by complementary studies examining implementation, including costs to time and direct ones (such as the unit costs of using a specific tool), potential harms, and how the tool’s risk scores can be linked to interventions or other changes to risk management. OxRec was introduced in the Netherlands to improve a previous system, which relied on older tools without OxRec’s distinct advantages. Although the findings of the first external validation study were promising, further evaluations as outlined above can examine whether the implementation of OxRec improves risk management and ultimately reduce criminal recidivism.
